# 107. Identification of state-level variables associated with changes in vaccination coverage rates in adults aged 18 to 64 in the United States

**DOI:** 10.1093/ofid/ofac492.185

**Published:** 2022-12-15

**Authors:** Amanda Eiden, Shannon Hunter, Diana Garbinsky, Mark A Price, Jon N Russo, Louise C Hartley, Mawuli Nyaku, Alexandra Bhatti

**Affiliations:** Merck, Philadelphia, Pennsylvania; RTI Health Solutions, Research Triangle Park, North Carolina; RTI Health Solutions, Research Triangle Park, North Carolina; RTI Health Solutions, Research Triangle Park, North Carolina; RTI-HS, Research Triangle Park, New Jersey; RTI Health Solutions, Research Triangle Park, North Carolina; Merck & Co., Doylestown, Pennsylvania; Merck & Co. Inc., east norriton, Pennsylvania

## Abstract

**Background:**

Despite the recognized value of life-course vaccination, adult vaccine uptake remains low. State-level vaccination coverage disparities may be associate with state-level variables (e.g., policies, programs, practices, and population characteristics). The objective of this study was to identify state-level variables associated with increased adult vaccination coverage rates (VCR) in the US for influenza, tetanus, herpes zoster (HZ), and pneumococcal vaccines over time.

**Methods:**

Retrospective, exploratory database analysis of 2011-2019 Behavioral Risk Factor Surveillance System (BRFSS) data was used to calculate state-level VCRs for adults 18-64 years old. Using publicly available data, state-level variables associated with increases in VCRs were identified via a systematic variable selection. The multivariable regression models included variables meeting the criteria: correlations > 0.4 or < -0.4 (continuous) or Kruskal-Wallis Test p-value < 0.2 (categorical).

**Results:**

Final multivariable regression models included 5 variables: Medicaid expansion status, accountable care organizations in place, health homes program, percentage of adults who report not seeing a doctor in past 12 months because of cost, and percentage of adults who report participating in any physical activity.

In the multivariable models, the following state-level variables were significantly associated with changes in VCR:

- Influenza: percentage of adults who report participating in any physical activity (p = .01)
- Pneumococcal: percentage of adults who report not seeing a doctor in the past 12 months because of cost (p = .02)
- HZ: health homes (p = .04); percentage of adults who report participating in any physical activity or exercise (p = .01); percentage of adults who report not seeing a doctor in past 12 months because of cost (marginally significant, p = .056)
- Tetanus: none

**Conclusion:**

Few state-level variables demonstrated an association with changes in VCR but some significant findings were observed in final models. Associated variables may not have a direct relationship but may be associated with public health infrastructure supporting vaccination ecosystems. Further research is underway to better understand the factors affecting adult vaccination.

**Disclosures:**

**Amanda Eiden, PhD, MBA, MPH**, Merck & Co., Inc.: Stocks/Bonds **Mark A. Price, MA, MEd**, Merck: Advisor/Consultant|Merck: I am an employee of RTI Health Solutions. Merck has contracted with my company to conduct the study described in the abstract. **Alexandra Bhatti, JD, MPH**, Merck & Co. Inc.: Grant/Research Support|Merck & Co. Inc.: Stocks/Bonds.

